# A Cumulative Framework for Identifying Overburdened Populations under the Toxic Substances Control Act: Formaldehyde Case Study

**DOI:** 10.3390/ijerph18116002

**Published:** 2021-06-03

**Authors:** Kristi Pullen Fedinick, Ilch Yiliqi, Yukyan Lam, David Lennett, Veena Singla, Miriam Rotkin-Ellman, Jennifer Sass

**Affiliations:** Natural Resources Defense Council, New York, NY 10011, USA; yiliqi@nrdc.org (I.Y.); ylam.pub@gmail.com (Y.L.); dlennett@nrdc.org (D.L.); vsingla@nrdc.org (V.S.); mrotkinellman@nrdc.org (M.R.-E.)

**Keywords:** multiple burdens, environmental justice, environmental policy, cumulative exposures, cumulative risk, pollution, community vulnerability, toxic chemicals, hazardous chemicals

## Abstract

Extensive scholarship has demonstrated that communities of color, low-income communities, and Indigenous communities face greater environmental and health hazards compared to communities with more White or affluent people. Low-income, Indigenous, Black, and/or other populations of color are also more likely to lack access to health care facilities, healthy food, and adequate formal education opportunities. Despite the mountains of evidence that demonstrate the existence and significance of the elevated toxic social and environmental exposures experienced by these communities, the inclusion of these factors into chemical evaluations has been scarce. In this paper, we demonstrate a process built with publicly available data and simple geospatial techniques that could be utilized by the U.S. Environmental Protection Agency (USEPA) to incorporate cumulative approaches into risk assessments under the Toxic Substances Control Act. The use of these approaches, particularly as they relate to identifying potentially exposed and susceptible subpopulations, would help USEPA develop appropriate risk estimates and mitigation strategies to protect disproportionately burdened populations from the adverse effects of chemical exposures. By utilizing such approaches to inform risk evaluation and mitigation, USEPA can identify and protect those most burdened and impacted by toxic chemicals, and finally begin to close the gap of environmental health inequities.

## 1. Introduction

Extensive scholarship has demonstrated that communities of color, low-income communities, and Indigenous communities face greater environmental and health hazards compared to communities with more White or affluent people. These communities disproportionately face extreme threats to their health from their environments, including high numbers of toxic “legacy” sites [1,2], large numbers and concentrations of chemical storage and industrial facilities [3,4], air pollution from traffic or ports [1,3,4,5], environmental exposure to heavy metals such as lead [6], workplace and take-home exposures [7,8,9], increased rates of drinking water violations [10], and heightened exposure to toxic chemicals, such as pesticides and other contamination in consumer products, food, and air [8,11,12,13]. The threats often converge in these communities due to historic and continuing racist and discriminatory policies and practices that perpetuate economic and health injustices, resulting in cumulative impacts on individual and population health [14,15].

Low-income, Indigenous, Black, and/or other populations of color are also more likely to lack access to health care facilities [16,17], healthy food [18,19,20], and adequate formal education opportunities [21]. Each of these are also associated with increases in risk of adverse health outcomes and can further compound the negative effects of hazardous environmental exposures on these populations [22]. It has been well-documented for many decades that significant disparities in health outcomes exist due to widespread social inequities [23]. In 1906, W.E.B. Du Bois noted that racial disparities in death from tuberculosis stemmed not from the biological, but the social [24]. In 1937, Wade Hampton Frost similarly noted that there were two factors most related to tuberculosis diagnoses—exposure and poverty [25]. Evidence of the relationships between health and social experiences—particularly experiences stemming from racism and poverty—has emerged for a wide range of health outcomes, including chronic diseases, communicable diseases, and injuries [26].

Despite the mountains of evidence that demonstrate the existence and significance of the elevated toxic social and environmental exposures experienced by these communities, the inclusion of these factors into chemical evaluations has been scarce [27]. Cumulative risk approaches, such as those that consider exposures to multiple, overlapping, chemical and non-chemical stressors have been recommended by the National Academies [28] and developed by USEPA [29]. Though cumulative risk approaches have had limited regulatory applications since USEPA released the Framework for Cumulative Risk Assessment in 2003 [29], the 2016 amendments to the Toxic Substances Control Act (TSCA) present a window of opportunity to integrate these methods into risk assessment processes for industrial, commercial, and consumer product chemicals. Additionally, Executive Order 13990 issued in January 2021—which instructs federal agencies to “advance environmental justice”, “limit exposure to dangerous chemicals”, and “hold polluters accountable, including those who disproportionately harm communities of color and low-income communities”—provides a timely impetus to integrate cumulative approaches across USEPA decisions [30].

The historical shortcomings of TSCA are well-known [31]. Passage of the 2016 amendments has led to substantially increased regulatory activity. For the first time in decades, USEPA is prioritizing chemicals for regulation, undertaking risk evaluations, finding unreasonable risks for the chemicals it has evaluated, and taking steps to restrict the use of these chemicals. While the number of chemicals to be evaluated is daunting, there is at least a process in motion for addressing some of the most toxic chemicals threatening public health.

In this paper, we discuss a pathway and process that could be utilized by USEPA to incorporate cumulative approaches into risk assessments under TSCA. The use of these approaches, particularly as they relate to identifying potentially exposed and susceptible subpopulations, would help USEPA develop appropriate risk estimates and mitigation strategies to protect disproportionately burdened populations from the adverse effects of chemical exposures. By utilizing a population health-based approach, that is, designing evaluations to protect the most impacted groups, USEPA can develop mitigation measures that are more protective of the entire population, including measures to further restrict chemical production and use that threatens public health.

## 2. Statutory Context

The principal federal law governing the safety of industrial, commercial, and consumer product chemicals is the Toxic Substances Control Act (TSCA). Under Section 6 of TSCA, the U.S. Environmental Protection Agency (USEPA) is required to conduct risk evaluations of priority chemicals, and where USEPA finds unreasonable risks arising from such chemicals, issue risk management rules to eliminate those risks.

Under Section 6(b)(4)(A) of the TSCA, USEPA’s risk evaluations must be conducted to “determine whether a chemical substance presents an unreasonable risk of injury to health or the environment, without consideration of costs or other non-risk factors, including an unreasonable risk to a potentially exposed or susceptible subpopulation identified as relevant to the risk evaluation by the Administrator, under the conditions of use. (Emphasis added)”.

TSCA defines “potentially exposed or susceptible subpopulations” (PESS) as “a group of individuals within the general population identified by the Administrator who, due to either greater susceptibility or greater exposure, may be at greater risk than the general population of adverse health effects from exposure to a chemical substance or mixture, such as infants, children, pregnant women, or the elderly [32]. (Emphasis added)”.

While the statute defines PESS based upon greater susceptibility or greater exposure, PESS identified based upon both criteria, such as cumulatively exposed subpopulations, warrant particular attention by USEPA for their potential to experience greater risk than the general population. Residents of fence-line communities (i.e., populations living in close proximity to polluting facilities) may often meet both criteria. People who work in polluting facilities and live near where they work may also meet both criteria.

### Identification of PESS under TSCA

**Figure 1:**
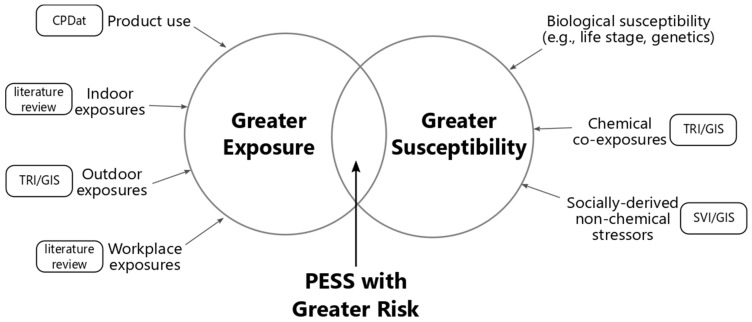
Conceptual framework for identifying potentially exposed and susceptible subpopulations to individual chemicals under the Toxic Substances Control Act. Rounded rectangles represent data sources/approaches used in this evaluation. Figure acronyms: USEPA Chemical and Products Database (CPDat), USEPA Toxics Release Inventory (TRI), geographic information system (GIS), Centers for Disease Control and Prevention Social Vulnerability Index (SVI).

When performing risk assessments under TSCA, USEPA is legally mandated to ensure that PESS with greater risk are considered in their evaluations. To do so, a crucial early step in the risk evaluation process should be to identify the populations that would be considered PESS (Figure 1). This identification process would enable USEPA to target the exposure scenarios of concern that should be included in the risk evaluation.

To identify populations with greater exposure to the chemical being evaluated, all exposure pathways for the chemical under evaluation should be considered, including exposures that result from the use of products containing the chemical and ambient air, drinking water, and other pathways arising from chemical production and disposal. To identify populations with greater susceptibility, USEPA should consider the multiple ways in which individuals and populations can have increased susceptibility to the adverse effects caused by the chemical, including intrinsic (e.g., biological) and acquired (e.g., environmental, behavioral, and nonchemical) factors [33].

Though there can be complicating factors in developing methods to evaluate the health risk resulting from the combination of chemical and nonchemical stressors [34], approaches to integrating PESS considerations do not require highly complex methods. In this case study, we develop a simple GIS-based approach with publicly available datasets to create an exposure profile of PESS that could be used under the TSCA. Our approach began with the selection of a chemical to evaluate, followed by an assessment of near- and far-field exposures to the chemical of concern. We then identified chemicals with a shared health endpoint to the chemical of concern and visualized the geographic areas in which co-exposures could occur. Finally, we integrated socially-derived non-chemical stressors (e.g., those that occur through the experience of inequitable social conditions) via inclusion of social vulnerability characteristics of geographic locations with overlapping chemical exposures (Table 1).

## 3. Case Study Demonstration with Formaldehyde

In December 2019, USEPA designated formaldehyde as a high-priority substance for risk evaluation under the amended TSCA, which began its formal assessment process. The first phase of the evaluation was the problem formulation/scoping phase, completed in August 2020 with the release of the final scoping document [35]. The scoping document will be used as the basis for the final risk evaluation of formaldehyde, which is anticipated to be completed in 2022.

Formaldehyde is a colorless, flammable gas that is present in the air, food, and manufactured products. Exposures to formaldehyde have been linked to a range of adverse health outcomes, including cancer and non-cancer effects, such as eye and nose irritation, asthma, reduced lung function, and memory impairments [36].

### 3.1. Methods and Approach

To identify PESS with greater risk associated with exposure to formaldehyde, we used data from the Toxics Release Inventory (TRI; USEPA) [37], Chemical and Products Database (CPDat; USEPA) [38], Integrated Risk Information System database (IRIS; USEPA) [39], California Safe Cosmetics Program Product Database (California Department of Public Health) [40], Homeland Infrastructure Foundation-Level Data (HIFLD; Department of Homeland Security) [41], and Social Vulnerability Index (SVI; Centers for Disease Control and Prevention) [42]. We chose TRI data as the primary data source for far-field exposure because of its greater generalizability to a larger number of chemicals than other data sources [e.g., the USEPA National Air Toxics Assessment (NATA) data [43]]. Though we only use TRI data for this assessment, USEPA should not limit its analysis to this source. For example, integrating the modeled ambient concentrations of Hazardous Air Pollutants, particularly those that can be formed in the atmosphere (including formaldehyde) can be helpful in identifying additional populations of concern.

To identify locations with formaldehyde emissions (and emissions of other chemicals of concern with a shared endpoint), we aggregated emitting facilities at the county-level and visualized results using qGIS. Buffer analysis for the co-locations of mobile home parks and formaldehyde-emitting facilities were also performed in qGIS.

To find potential relationships between social vulnerability factors and emissions of formaldehyde, Pearson correlations were performed using R to identify the strength, direction, and statistical significance. Correlation coefficients above 0.1 or below −0.1 with *p*-values less than 0.05 were considered indicative of potential relationships.

### 3.2. Identification of Populations with Potential for Greater Exposure to Formaldehyde

Individuals and populations can be exposed to chemicals by routes including oral, dermal, and inhalation, and via a number of sources, including environmental media (e.g., air, soil, water), food, and consumer products. To identify PESS, we considered exposures from both near- and far-field sources.

#### 3.2.1. Increased Exposure to Formaldehyde from Far-Field Exposures

While indoor air environments represent the largest source of exposures to formaldehyde generally, outdoor (far-field) exposures, particularly in areas with sizeable industrial production/uses, can contribute significantly to population-level risk. People living in locations with significant localized releases of formaldehyde may face greater exposure than the general population and should be considered candidates for PESS.

**Figure 2:**
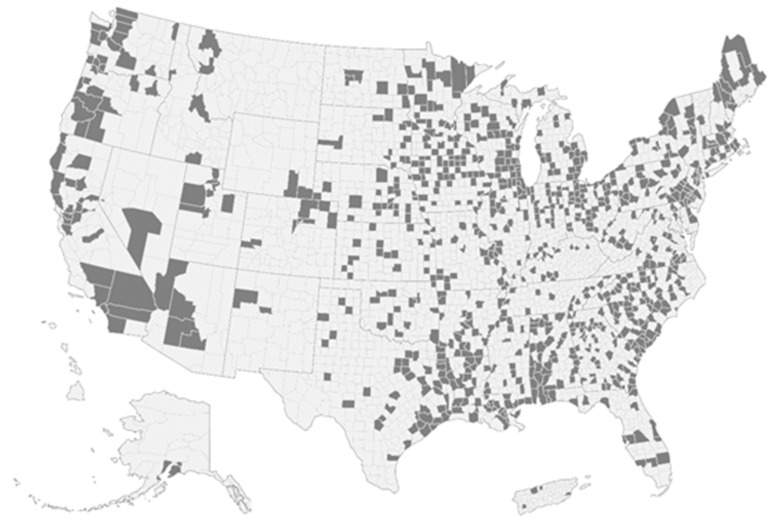
Counties (in grey) with facility-level emissions of formaldehyde between 2000 and 2018 (TRI).

Using emissions data from the USEPA Toxics Release Inventory (TRI), we identified 1572 facilities across 858 counties with formaldehyde emissions between 2000 and 2018 (Figure 2). Given the long latency period for cancer (the common health endpoint we use in this case study), we chose a long timeframe to capture historic exposures that could contribute to present-day and future adverse health outcomes. Though a multi-decadal timeframe may not be necessary for every chemical, latency periods and exposure pathways (e.g., air, soil) should be considered when identifying potential far-field sources of exposure.

#### 3.2.2. Increased Exposure to Formaldehyde from Near-Field Exposures

In addition to far-field exposures to formaldehyde, many expert assessments from the U.S. [36] and globally [44] have documented high indoor (near-field) air concentrations of formaldehyde—including cases in which indoor concentrations exceed outdoor levels. Cigarette smoke [44,45,46], off-gassing of plywood and composite wood products [47,48], urea formaldehyde foam insulation (UFFI) [49,50], carpets and textiles [51,52], varnishes [53], and consumer products such as cleaners and polishers [51,54] can all be significant sources of near-field exposures to formaldehyde. Indoor air in mobile homes has been found to be as high as 5000 ppb—far above the normal and polluted air pollution ranges of ~20–100 ppb and 100–250 ppb, respectively.

To identify potential sources of formaldehyde in homes, we utilized data from the USEPA Chemical and Products Database (CPDat) [38] and the California Safe Cosmetics Program Product Database [40]. Our analysis found over 1,100 unique products from a broad range of industry and categories that contain formaldehyde—including manufacturing, agricultural, and personal care products (see Supplementary Table S1). Of the personal care products category, we found products ranging from hand soaps to lotions to products marketed specifically to Black women [8]. When developing exposure estimates for PESS under TSCA, USEPA should identify those populations that have greater exposure from all pathways (aggregate exposures), including near- and far-field exposures.

### 3.3. Identification of Populations with Increased Susceptibility to Formaldehyde

In identifying and accounting for greater susceptibility in risk evaluations under TSCA, USEPA should take into account both intrinsic and acquired factors. Intrinsic susceptibility includes factors like life stage, genetics, and gender. Acquired susceptibility can arise from the experience of social disadvantage (e.g., racial and economic discrimination) and can include factors such as lack of access to health care, heightened psychosocial stress, pre-existing disease, inadequate nutrition, tobacco and alcohol use, and heightened exposure to harmful chemicals in the environment [33]. While intrinsic factors (e.g., genetics) have been interrogated with more frequency in risk assessment [55], the consideration and inclusion of acquired susceptibility factors has been virtually non-existent despite the more important role they may play in increasing environmental health disparities [27].

#### 3.3.1. Increased Susceptibility to the Health Risk of Formaldehyde Exposures Due to Co-Exposures to Chemicals with Similar Health Endpoints

Cumulative exposure assessment evaluates the combined exposure to chemical and non-chemical stressors via multiple pathways that can lead to an adverse outcome (e.g., cancer). While there are many ways to group chemicals, for this case study we grouped chemicals according to a shared health endpoint. This approach was recommended in the 2008 National Academies report on ‘Phthalates and Cumulative Risk Assessment’ [56]. Although formaldehyde is a multi-site carcinogen, and also causes non-cancer adverse health effects, including urinary system toxicity and gastrointestinal system toxicity, we selected a single health endpoint—respiratory cancer—to demonstrate one possible way to group similarly acting chemicals. We chose this endpoint because it is well-supported by authoritative bodies, including the U.S. Department of Health and Human Services (HHS) that lists formaldehyde as known to cause cancer in people, based on several types of cancers, most predominantly of the nose and throat (nasal, nasopharyngeal, and respiratory systems) [57]. While we chose respiratory cancer for illustration purposes only, USEPA must consider the most sensitive endpoints for risk evaluations under TSCA, including both cancer and non-cancer endpoints. USEPA should also consider exposures to other chemicals with shared health endpoints to which PESS may be exposed.

Using respiratory cancer as our health endpoint, we searched for known respiratory carcinogens in the USEPA Integrated Risk Information System (IRIS) public database. IRIS assessments provide robust, peer-reviewed, and publicly available hazard characterizations and dose-response assessments for cancer and non-cancer outcomes [39,58]. They are produced with consideration of the needs of TSCA and other USEPA programs [59]. While IRIS assessments are not regulations, they are used by regulators—at the USEPA, by local, state, and regional governments, and around the world—to set health-based standards for chemicals in air, water, food, or soil. We searched the IRIS database using the “Advanced Search” function using “cancer” as the endpoint, “inhalation” and “oral” for routes of exposure, and “respiratory” for the system affected. Our search identified a total of 24 chemicals (out of a total number of chemicals of 485), including formaldehyde, listed by USEPA as linked to an elevated risk of respiratory cancer by either the oral or inhalation routes of exposure (see Supplementary Table S2) [60].

To identify populations with heightened risk for adverse health outcomes due to co-exposure to formaldehyde and other respiratory carcinogens, we used TRI data for the 16 IRIS-listed respiratory carcinogens with information in the database (including formaldehyde). The 15 chemicals in addition to formaldehyde were acetylaldehyde, acrylonitrile, inorganic arsenic, asbestos, benzotrichloride, beryllium, bis(chloromethyl)ether (BCME), cadmium, chloroprene, 1,2-dibromoethane, dichloromethane, 1,4-dioxame, epichlorohydrin, hydrazine, and propylene oxide.

**Figure 3:**
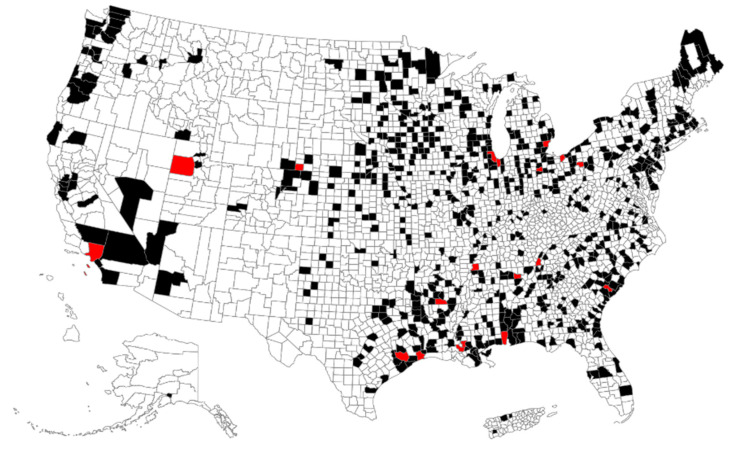
Counties (in black) with facility-level emissions between 2000 and 2018 (TRI) of formaldehyde and one or more respiratory carcinogens identified in the USEPA IRIS database. Counties shown in red have facility-level emissions of formaldehyde and nine or more IRIS-assessed respiratory carcinogens.

Our analysis found 647 counties across the country that had facility-level emissions of formaldehyde and at least one of the IRIS respiratory carcinogens. There were 19 counties (shown in red in Figure 3) that had facility-level emissions of formaldehyde and nine or more of the IRIS respiratory carcinogens for a total number of respiratory carcinogens ranging from 10–16.

#### 3.3.2. Increased Susceptibility to Formaldehyde Due to Co-Exposures to Socially-Derived Nonchemical Stressors

Low-income, Black, Indigenous, and/or other populations of color can suffer from multiple, population-level-acquired susceptibility factors. These communities shoulder disproportionately high levels of exposure to hazardous chemicals from multiple pollution sources and through numerous exposure pathways [12,13]. For example, overburdened communities face increased rates of contaminated drinking, measured by water violations [10].

Sustained exposure to negative social forces can increase the occurrence of psychosocial stress, which can influence susceptibility to chemical stressors [34]. The negative effects of chronic adverse social experiences (e.g., racial and economic discrimination) can alter key physiological systems (e.g., immune, cardiovascular, and endocrine systems) via a range of biological pathways, including increased inflammatory responses [61] and epigenetic changes [62]. These physiological and biological responses to stress can amplify the occurrence of a range of disease states [63,64,65]. To ensure the proper identification of PESS under TSCA, it is therefore of paramount importance to account for social characteristics that can lead to increased susceptibility—particularly in places where exposure and socially-derived susceptibility overlap.

To determine if formaldehyde exposure was heightened for populations with a high potential for social disadvantage, we combined formaldehyde emissions data from TRI (from 2000–2018) with the Centers for Disease Control and Prevention Social Vulnerability Index (SVI). We performed exploratory statistical assessments of the potential associations between formaldehyde air emissions and socially derived non-chemical stressors via Pearson correlations.

Our exploratory assessment revealed statistically significant (*p* < 0.05), positive correlations between the number of facilities emitting formaldehyde (between 2000 and 2018) and estimated percentages of the population designated as single-parent households, classified as a minority (i.e., all persons except White, non-Hispanic), over the age of 5 that spoke English “less than well”, and housing in structures with 10 or more units. We found statistically significant (*p* < 0.05) negative correlations between estimated percentages of the population that were aged 65 and older, civilian noninstitutionalized populations with a disability, and a percentage of mobile homes. Additionally, we found positive, statistically significant associations (*p* < 0.05) between the number of facilities and two SVI themes—“Minority Status/Language” and “Housing Type and Transportation”.

While these correlation coefficients are modest (Table 2), they suggest that socially-derived non-chemical stressors should be accounted for in the risk evaluation of formaldehyde under the TSCA.

#### 3.3.3. Specific Characteristics of PESS in Areas with Increased Exposure and Susceptibility to Formaldehyde

Our national analysis found several counties/parishes in Arkansas (Union), Tennessee (Shelby), Texas (Harris), and Louisiana (Ascension, East Baton Rouge, and Iberville) that could provide representative snapshots of PESS under the TSCA. Though our focus was on the general public in these locations, people that work in formaldehyde facilities and live in communities adjacent to emitting facilities are even more exposed, and thus should be evaluated by USEPA accordingly.

Our analysis found residents of these counties were more likely to have: (1) elevated exposure to formaldehyde in the outdoor air because of a high concentration of production facilities, (2) combined exposures to other chemicals with similar adverse health impacts in the outdoor air, (3) elevated cancer risk resulting from combined exposure to formaldehyde and respiratory carcinogens, and (4) increased susceptibility to illness and disease resulting from formaldehyde exposure due to socially-derived non-chemical stressors.

**Figure 4:**
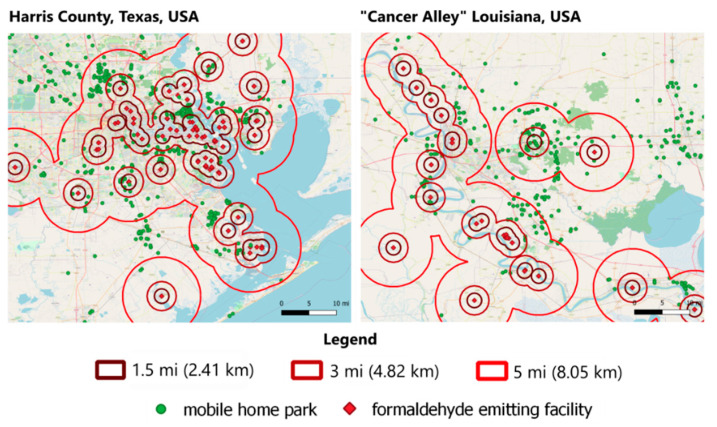
Density of mobile home parks near facilities releasing formaldehyde (between 2000–2018, USEPA TRI) around Harris County, TX and in the I-10 corridor between New Orleans and Baton Rouge, LA—so-called “Cancer Alley” Parishes.

In addition to the county-level analyses, we also assessed the numbers and proximity of mobile home parks to formaldehyde-emitting facilities (see Figure 4). Interestingly, though our national analysis indicated a negative correlation between the estimated percentages of mobile homes, a localized analysis revealed a different picture. Using TRI data and the Homeland Infrastructure Foundation-Level Data (HIFLD) [41] from the Department of Homeland Security, we found nearly 2000 mobile home parks within 1 mile of facilities that emitted formaldehyde between 2000 and 2018. We found 5427 mobile home parks within 3 miles of formaldehyde-emitting facilities and 15,808 mobile home parks within 5 miles of formaldehyde-emitting facilities. This finding reveals the importance of accounting for the local conditions that can increase exposures and susceptibilities to the chemical being evaluated.

Populations living in mobile home parks near formaldehyde-emitting facilities, particularly in areas with high levels of other chemicals with shared adverse health endpoints, may experience high levels of formaldehyde exposure from both ambient and indoor air. These populations, especially children, pregnant women, and workers, are particularly important to identify and account for in places with high burdens due to other chemical and non-chemical stressors.

## 4. Discussion

In this paper, we presented an approach, using formaldehyde as a case study, that identifies populations with increased exposure and susceptibility to formaldehyde. We used publicly available datasets from state and local agencies to develop a model of populations and environmental conditions that could serve as a guidepost for USEPA in developing protective chemical assessments. Our approach might also be useful in prioritizing chemicals. For example, USEPA might seek to prioritize some chemicals causing disproportionate impacts in fenceline communities and use an approach similar to ours to identify those chemicals [66].

This risk-based effort complements efforts to conduct place-based cumulative impact analyses and incorporate such analyses into siting and permitting decisions. While both types of approaches draw on spatial, environmental, and health data, the latter seeks to impose restrictions on introducing, expanding, or renewing facilities that emit pollution in communities that are already disproportionately burdened by cumulative impacts [67,68,69]. The approach we present here also seeks to protect overburdened populations by accounting for the impacts/presence of multiple sources of pollution, but does so in the context of regulating national chemical production and uses.

While our assessment was limited in some areas due to the lack of available data (e.g., TRI emissions data for only 16 of the 24 of the IRIS-assessed respiratory carcinogens), USEPA has the authority to obtain and publish the emissions data for these chemicals and should include the resulting exposures in its risk evaluation. Local ambient air monitoring in fenceline communities could also contribute important exposure data.

Additionally, though our analysis generally focused on the PESS in the general population, worker exposures—particularly those that live in close proximity to polluting facilities—should be high-priority populations to evaluate for TSCA risk assessments. Workplace health and safety hazards for low-wage jobs may also cause “take-home” exposures to family members (for example, agricultural workers [7], first responders, salon workers [8], and custodial workers [9]).

This case study was also limited in its consideration of the range on factors for both intrinsic and acquired susceptibility. Though our approach was focused on chemically- and socially-acquired (via social vulnerability) susceptibility, other highly relevant life stages (e.g., age and pregnancy status) and acquired pathways (e.g., rates of pre-existing disease) should also be considered. As we demonstrated in our mobile homes finding, data at the local scale, particularly health data, could be particularly important and informative.

Finally, while this paper used respiratory cancer as the unifying health endpoint for identifying chemicals with the potential to increase susceptibility, USEPA should not limit itself to this particular endpoint. For example, non-cancer health effects, such as asthma [70], gastrointestinal toxicity, and urinary system toxicity should also be included, along with additional chemicals that cause the same effects and to which PESS populations may also be exposed. In the scoping phase of a TSCA evaluation, USEPA is required to specify the hazards, exposures, conditions of use, and the PESS it expects to consider in the risk evaluation. Thus, USEPA should describe all adverse health outcomes to be considered as part of the risk evaluation, to ensure that PESS will be adequately identified and protected. Developing real-world, cumulative exposure assessments on the health endpoints of greatest concern, including the influence of chemical and non-chemical stressors, should be a standard practice in TSCA risk assessments.

## 5. Conclusions

To accurately evaluate the health risk from chemicals and to ensure the incorporation of Executive Order 13990 into TSCA implementation, USEPA must use a cumulative framework to identify the PESS at greatest risk from exposure to priority chemicals. This includes combined exposures for these populations through all pathways—ingestion, inhalation, and dermal absorption—and from all sources of exposure, including diet, workplace, and consumer products, and the ambient environment. USEPA should consider additional chemical exposures, as well as non-chemical stressors, particularly those associated with race and poverty using a cumulative framework. By utilizing such approaches to inform risk evaluation and mitigation, USEPA can identify and then protect those most burdened and impacted by toxic chemicals, and finally begin to close the gap of environmental health inequities.

## Figures and Tables

**Table 1:** GIS-based approach to identify and account for PESS in TSCA risk evaluations.

Step	Description
1	Identify chemical of concern being evaluated
2	a. Determine geographic locations with potential for far-field exposure. Sources of geographic information include peer-reviewed literature, chemical release databases (e.g., Toxic Release Inventory), modeled exposure databases (e.g., National Emissions Inventory).
	b. Identify possible sources of near-field exposures. Sources of information include peer-reviewed literature, grey literature, product databases (e.g., Chemical and Products Database), authoritative assessments (e.g., Integrated Risk Information System assessments).
3	a. Identify most sensitive endpoints for chemical being evaluated. This step is often performed during the risk evaluation scoping phase and can include peer-reviewed literature, grey-literature, authoritative evaluations, and other information sources.
	b. Identify chemicals with common health endpoint. Sources of data include peer-reviewed literature, grey literature, authoritative assessments (e.g., Integrated Risk Information System), toxicological databases (e.g., ToxCast).
	c. Determine geographic locations with overlap between chemical of concern and other chemicals with shared endpoint. Sources of geographic information include peer-reviewed literature, chemical release databases (e.g., Toxic Release Inventory), modeled exposure databases (e.g., National Emissions Inventory).
4	a. Identify relevant non-chemical stressors to be accounted for in assessment. Sources of information include peer-reviewed literature, reports, and datasets with sociodemographic indicators (e.g., American Community Survey, Social Vulnerability Index).
	b. Assess the overlap of chemical and non-chemical stressors for geographic hotspots (i.e., areas with co-exposures to multiple chemicals associated with shared adverse health outcome).
5	Develop profile of populations with greater exposure and/or susceptibility to be used in risk characterization and management strategies.

**Table 2:** Pearson correlations between formaldehyde-emitting facilities and Social Vulnerability Index (SVI) variables with *p* values < 0.05.

SVI 2018 Variable Name	SVI 2018 Variable Description	Number of Formaldehyde Emitting Facilities in 2000
EP_PCI	Per capita income estimate, 2014–2018 ACS	0.122752422
EP_NOHSDP	Percentage of persons with no high school diploma (age 25+) estimate	−0.049584352
EP_AGE65	Percentage of persons aged 65 and older estimate, 2014–2018 ACS	−0.169812317
EP_AGE17	Percentage of persons aged 17 and younger estimate, 2014–2018 ACS	0.052260265
EP_DISABL	Percentage of civilian noninstitutionalized population with a disability estimate, 2014–2018 ACS	−0.1451448
EP_SNGPNT	Percentage of single parent households with children under 18 estimate, 2014–2018 ACS	0.111258637
EP_MINRTY	Percentage minority (all persons except white, non-Hispanic) estimate, 2014–2018 ACS	0.132175661
EP_LIMENG	Percentage of persons (age 5+) who speak English “less than well” estimate, 2014–2018 ACS	0.110177734
EP_MUNIT	Percentage of housing in structures with 10 or more units estimate	0.249835074
EP_MOBILE	Percentage of mobile homes estimate	−0.151929548
RPL_THEME3	Percentile ranking for Minority Status/Language theme	0.185509699
RPL_THEME4	Percentile ranking for Housing Type/Transportation theme	0.109942915

## Data Availability

Data sources used and cited in this article are publicly available from the USEPA (TRI [37], CPDat [38], IRIS [39]) California Department of Public Health (California Safe Cosmetics Program Product Database [40]), Department of Homeland Security (HIFLD [41]) and Centers for Disease Control and Prevention (SVI [42]).

## Supplementary Materials

The following are available online at https://www.mdpi.com/article/10.3390/ijerph18116002/s1, Table S1: Products cassettes identified in CPDat with at least 10 unique products containing formaldehyde, Table S2: IRIS classified respiratory carcinogens.
