# Supplementary material for: A Cumulative Framework for Identifying Overburdened Populations under the Toxic Substances Control Act: Formaldehyde Case Study
Source: Int J Environ Res Public Health. 2021 Jun 3;18(11):6002. doi: 10.3390/ijerph18116002 (PMC8199872; doi:10.3390/ijerph18116002)
Supplement: Supplementary file 1 [file ijerph-18-06002-s001.zip › ijerph-1225560-supplementary.pdf]

TITLE: A Cumulative Framework for Identifying Overburdened Populations under the Toxic Substances Control Act: Formaldehyde Case Study

Kristi Pullen Fedinick*, Ilch Yiliqi, Yukyan Lam, David Lennett, Veena Singla, Miriam Rotkin-Ellman, Jennifer Sass*

Supplemental Material

Table S1. Products cassettes identified in CPDat [2] with at least 10 unique products containing formaldehyde

**Product cassettes**	Number of unique products
Manufacturing[1]	325
Personal care products	68
Pesticide[2]	67
Paint	59
Building material	41
Surface treatment	38
Cleaning washing	38
Adhesive	33
Fluid property modulator	25
Printing	22
Building construction	22
Industrial	20
Facility	19
Agricultural	18
Resource extraction	17
Home maintenance	15
Construction	11
Colorant	11
Water treatment	11
Polish	11
Photographic	10
Automotive care	10

Data downloaded from https://comptox.epa.gov/dashboard/dsstoxdb/results?search=DTXSID7020637#product-use-categories on May 28, 2020.

[1] Products in this cassette include metals (like iron and steel), building materials (like wood), machines (like agricultural, medical, electrical and forestry machines), food (like dairy, meat, grain, fruit, cooking oils, vegetable, fats, cheese and fish), chemical, plastics, furniture, electrical, textile, apparel, leather, rubber, oil, automotive, cigarettes, fertilizer, and pesticides etc. Note that some of these product categories are also listed as a separate cassette.

[2] Products in this cassette include antimicrobial, biocide, food additive, insect repellent, bactericide, fungicide, herbicide etc.

Products identified in "personal care products" cassette covers a wide range of personal care and cosmetic products, including the following products consumers use regularly:

- Hand wash
- Body wash and shower gel
- Shampoo
- Lotion (body lotion, hand lotion, and foot lotion)
- Hair styling products
- Hair dye
- Face cream and moisturizer
- Face mask
- Foundation and concealer
- Eye makeup and tools
- Nail products
- Fragrance and perfume

Table S2. IRIS classified respiratory carcinogens [1]

CHEMICAL NAME (CAS#)	BASIS OF SLOPE FACTOR	CLASSIFICATION	TUMOR SITE	TUMOR TYPE	SLOPE FACTOR
**Acetaldehyde (75-07-0)**	Inhalation	B2 (Probable human carcinogen - based on sufficient evidence of carcinogenicity in animals) (1986 guidelines)	Respiratory	Nasal squamous cell carcinoma or adenocarcinoma	2.2×10^{-6} per µg/m^3
**Acrylonitrile 107-13-1**	Inhalation	B1 (Probable human carcinogen - based on limited evidence of carcinogenicity in humans) (1986 guidelines)	Respiratory	Respiratory cancer	6.8×10^{-5} per µg/m^3
**Arsenic, Inorganic (7440-38-2)**	Inhalation	A (Human carcinogen) (1986 guidelines)	Respiratory	Lung cancer	4.3×10^{-3} per µg/m^3
**Asbestos (1332-21-4)**	Inhalation	A (Human carcinogen) (1986 guidelines)	Respiratory	Lung cancer and mesothelioma	2.3×10^{-1} per f/mL
**Benzotrichloride (98-07-7)**	Oral	B2 (Probable human carcinogen - based on sufficient evidence of carcinogenicity in animals) (1986 guidelines)	Respiratory	Lung, adenocarcinoma	1.3×10^{1} per mg/kg-day

Table S2. IRIS classified respiratory carcinogens [1]

CHEMICAL NAME (CAS#)	BASIS OF SLOPE FACTOR	CLASSIFICATION	TUMOR SITE	TUMOR TYPE	SLOPE FACTOR
**Beryllium and compounds** **(7440-41-7)**	Inhalation	B1 (Probable human carcinogen - based on limited evidence of carcinogenicity in humans) (1986 guidelines) Carcinogenic potential cannot be determined (1996 guidelines) Known/likely human carcinogen (1996 guidelines)	Respiratory	Lung cancer	2.4×10^{-3} per µg/m^3
**Bis(chloromethyl)ether (BCME)** **(542-88-1)**	Inhalation	A (Human carcinogen) (1986 guidelines)	Respiratory	Respiratory tract tumors	6.2×10^{-2} per µg/m^3
**Cadmium** **(7440-43-9)**	Inhalation	B1 (Probable human carcinogen - based on limited evidence of carcinogenicity in humans) (1986 guidelines)	Respiratory	Lung, trachea, bronchus cancer deaths	1.8×10^{-3} per µg/m^3
**Chloroprene** **(126-99-8)**	Inhalation	Likely to be carcinogenic to humans (2005 guidelines)	Respiratory	alveolar/ bronchiolar adenoma or carcinoma hemangioma/ hemangiosarcoma (all organs) mammary gland adenocarcinoma, carcinoma, or adenoacanthoma forestomach squamous cell papilloma or carcinoma hepatocellular adenoma or carcinoma Harderian gland adenoma or carcinoma skin sarcoma and	3×10^{-4} per µg/m^3

**Table S2. IRIS classified respiratory carcinogens** [1]					
CHEMICAL NAME (CAS#)	BASIS OF SLOPE FACTOR	CLASSIFICATION	TUMOR SITE	TUMOR TYPE	SLOPE FACTOR
				Zymbal's gland carcinoma	
**1,2-Dibromoethane (106-93-4)**	Inhalation	Likely to be carcinogenic to humans (1999 guidelines)	Respiratory	Nasal cavity (includes adenoma, adenocarcinoma, papillary adenoma, squamous cell carcinoma, and or/papilloma), hemangiosarcomas, mesotheliomas	$3x10^{-4}$ per µg/m^3 (central tendency estimate)
**Dichloromethane (75-09-2)**	Inhalation	Likely to be carcinogenic to humans (2005 guidelines)	Respiratory	Hepatocellular carcinomas or adenomas, bronchoalveolar carcinomas or adenomas	$1x10^{-8}$ per µg/m^3
**1,4-Dioxane (123-91-1)**	Inhalation	Likely to be carcinogenic to humans (2005 guidelines)	Respiratory	Multiple (nasal, liver, kidney, peritoneal, mammary gland, and Zymbal gland)	$5x10^{-6}$ per µg/m^3
**Epichlorohydrin (106-89-8)**	Inhalation	B2 (Probable human carcinogen - based on sufficient evidence of carcinogenicity in animals) (1986 guidelines)	Respiratory	Nasal cavity tumors	$1.2x10^{-6}$ per µg/m^3
**Formaldehyde (50-00-0)**	Inhalation	B1 (Probable human carcinogen - based on limited evidence of carcinogenicity in humans) (1986 guidelines)	Respiratory	Squamous cell carcinoma	$1.3x10^{-5}$ per µg/m^3
**Hydrazine/Hydrazine sulfate (302-01-2)**	Inhalation	B2 (Probable human carcinogen - based on sufficient evidence of carcinogenicity in	Respiratory	Nasal cavity adenoma or adenocarcinoma	$4.9x10^{-3}$ per µg/m^3

Table S2. IRIS classified respiratory carcinogens [1]					
CHEMICAL NAME (CAS#)	BASIS OF SLOPE FACTOR	CLASSIFICATION	TUMOR SITE	TUMOR TYPE	SLOPE FACTOR
		animals) (1986 guidelines)			
**Propylene oxide (75-56-9)**	Inhalation	B2 (Probable human carcinogen - based on sufficient evidence of carcinogenicity in animals) (1986 guidelines)	Respiratory	Nasal cavity hemangioma or hemangiosarcoma	3.7×10^{-6} per µg/m^3
**IRIS respiratory carcinogens not included in the geographic assessment due to lack of inclusion in the TRI**					
**1,3-Dichloropropene (542-75-6)**	Inhalation	B2 (Probable human carcinogen - based on sufficient evidence of carcinogenicity in animals) (1986 guidelines) Known/likely human carcinogen (1996 guidelines)	Respiratory	Bronchioalveolar adenoma	4×10^{-6} per µg/m^3
**Benzo[a]pyrene (BaP) (50-32-8)**	Inhalation	Carcinogenic to humans (2005 guidelines)	Respiratory	Squamous cell neoplasia in the larynx, pharynx, trachea, nasal cavity, esophagus, and forestomach.	6×10^{-4} per µg/m^3
**Chromium(VI) (18540-29-9)**	Inhalation	A (Human carcinogen) (1986 guidelines) Carcinogenic potential cannot be determined (1996 guidelines) D (Not classifiable as to human carcinogenicity) (1986 guidelines) Known/likely human carcinogen (1996 guidelines)	Respiratory	Lung cancer	1.2×10^{-2} per µg/m^3

Table S2. IRIS classified respiratory carcinogens [1]

CHEMICAL NAME (CAS#)	BASIS OF SLOPE FACTOR	CLASSIFICATION	TUMOR SITE	TUMOR TYPE	SLOPE FACTOR
**Coke oven emissions**	Inhalation	A (Human carcinogen) (1986 guidelines)	Respiratory	Respiratory cancer	$6.2x10^{-4}$ per µg/m^3
**Hexahydro-1,3,5-trinitro-1,3,5-triazine (RDX)** **(121-82-4)**	Oral	Suggestive evidence of carcinogenic potential (2005 guidelines)	Respiratory	Liver (hepatocellular adenomas or carcinomas) and lung (alveolar/bronchiolar adenomas or carcinomas)	$8x10^{-2}$ per mg/kg-day
**Libby Amphibole asbestos**	Inhalation	Carcinogenic to humans (2005 guidelines)	Respiratory	Cancer mortality from lung cancer and mesothelioma	$1.7x10^{-1}$ per fiber/cc
**Nickel refinery dust**	Inhalation	A (Human carcinogen) (1986 guidelines)	Respiratory	Lung cancer	$2.4x10^{-4}$ per µg/m^3
**Nickel subsulfide** **(12035-72-2)**	Inhalation	A (Human carcinogen) (1986 guidelines)	Respiratory	Lung cancer	$4.8x10^{-4}$ per µg/m^3

References:

1. Chemical Search | IRIS | US EPA https://cfpub.epa.gov/ncea/iris/search/basic/index.cfm (accessed May 11, 2020).

2. USEPA Office of Research and Development. Chemical and Products Database (CPDat) https://www.epa.gov/chemical-research/chemical-and-products-database-cpdat (accessed Apr 27, 2021).
